# High Dengue Case Capture Rate in Four Years of a Cohort Study in Nicaragua Compared to National Surveillance Data

**DOI:** 10.1371/journal.pntd.0000633

**Published:** 2010-03-16

**Authors:** Katherine Standish, Guillermina Kuan, William Avilés, Angel Balmaseda, Eva Harris

**Affiliations:** 1 Sustainable Sciences Institute, Managua, Nicaragua; 2 Centro de Salud Sócrates Flores Vivas, Managua, Nicaragua; 3 Departamento de Virología, Centro Nacional de Diagnóstico y Referencia, Ministry of Health, Managua, Nicaragua; 4 Division of Infectious Diseases, School of Public Health, University of California, Berkeley, California, United States of America; Pediatric Dengue Vaccine Initiative, United States of America

## Abstract

Dengue is a major public health problem in tropical and subtropical regions; however, under-reporting of cases to national surveillance systems hinders accurate knowledge of disease burden and costs. Laboratory-confirmed dengue cases identified through the Nicaraguan Pediatric Dengue Cohort Study (PDCS) were compared to those reported from other health facilities in Managua to the National Epidemiologic Surveillance (NES) program of the Nicaraguan Ministry of Health. Compared to reporting among similar pediatric populations in Managua, the PDCS identified 14 to 28 (average 21.3) times more dengue cases each year per 100,000 persons than were reported to the NES. Applying these annual expansion factors to national-level data, we estimate that the incidence of confirmed pediatric dengue throughout Nicaragua ranged from 300 to 1000 cases per 100,000 persons. We have estimated a much higher incidence of dengue than reported by the Ministry of Health. A country-specific expansion factor for dengue that allows for a more accurate estimate of incidence may aid governments and other institutions calculating disease burden, costs, resource needs for prevention and treatment, and the economic benefits of drug and vaccine development.

## Author Summary

National public health and epidemiology programs are often tasked with tracking certain infectious diseases, but many barriers lead to under-reporting. In tropical and subtropical countries where dengue fever is endemic, under-reporting may be due to misdiagnosis, limitations of the WHO case classification, and lack of laboratory infrastructure or resources, among other issues. In Nicaragua's capital, Managua, we compared the number of dengue cases identified in a cohort study of 2- to 12-year-old children in one health center to the number of pediatric cases reported from all other municipal health centers in Managua to the National Epidemiologic Surveillance (NES) program. In the years 2004–2008, between 13 and 65 dengue cases were identified in the cohort (approximately 3,700 participants), and between 0 and 51 cases were reported by individual health centers in Managua. When the incidence per 100,000 people was calculated and compared, an average of 21 times more dengue cases were identified in the cohort study compared to the number reported to NES. Application of such an expansion factor may help governments and other health institutions to estimate the actual number of dengue cases in a population and therefore better allocate resources for prevention, treatment, and drug and vaccine development.

## Introduction

Dengue is a mosquito-borne illness that is a major public health problem in tropical and subtropical regions, causing millions of cases annually [1]. However, under-reporting of dengue cases to national surveillance systems hinders accurate local, regional and global calculations of disease burden and costs, which in turn impact appropriate resource allocation and availability of reliable data for vaccine and drug development. Under-reporting has been attributed to a range of factors, including misdiagnosis, limited laboratory capabilities, poor application of the World Health Organization (WHO) case definition, and limitations of national reporting systems, among others [2],[3]. Furthermore, scarce data on inapparent dengue virus (DENV) infections limits estimates of risk for more severe secondary infections. To address the lack of a country-specific estimate of disease burden based on surveillance data, we calculated the difference in dengue case capture rates between a pediatric dengue cohort study (PDCS) and the Ministry of Health dengue surveillance program (“expansion factor”) in Managua, Nicaragua, which can be applied to national statistics for more accurate estimations of dengue burden.

## Materials and Methods

The PDCS is a community-based, prospective cohort study that was initiated in 2004 in a low-to-middle-income district of Managua and is based in a municipal clinic, the Health Center Sócrates Flores Vivas (HCSFV), which is the principal source of health care for the district's population. The study captured possible dengue cases through “enhanced” passive surveillance by study physicians and nurses at the HCSFV and periodic home visits for follow-up and monitoring of study compliance [4]. Cohort participants, initially aged 2–9 years old in 2004, were followed closely for all illnesses, and children who presented with fever were screened for signs and symptoms of dengue. Those who met WHO criteria for suspected dengue (acute febrile illness with two or more of the following symptoms or signs: headache, retro-orbital pain, myalgia, arthralgia, rash, hemorrhagic manifestations, leukopenia, or platelets ≤150,000/mm^3^) as well as those with undifferentiated fever were evaluated for acute DENV infection. A dengue case was considered laboratory-confirmed when 1) DENV was isolated, 2) DENV RNA was demonstrated by reverse-transcriptase polymerase chain reaction (RT-PCR), 3) seroconversion was observed with paired acute and convalescent phase sera by IgM capture ELISA or Inhibition ELISA [5],[6], or 4) a ≥4-fold increase in antibody titer in paired acute and convalescent sera was observed by Inhibition ELISA [7]. Overall, 118 (74%) of the confirmed DENV infections in the PDCS complied with the WHO case definition (15 (88%), 44 (68%), 8 (62%), and 51 (80%) in years 2004–5, 2005–6, 2006–7, 2008–9, respectively), while 41 (26%) had undifferentiated fever (2 (12%), 21 (32%), 5 (38%), and 13 (20%) in years 2004–5, 2005–6, 2006–7, 2007–8, respectively) (8). There were 2, 2, 1, and 16 hospitalized cases in the cohort in 2004–5, 2005–6, 2006–7, 2007–8, respectively, all of which were compliant with the WHO case definition.

Inapparent DENV infections, presented here separately from symptomatic cases, were identified through serological testing of paired annual blood draws from healthy subjects, which were collected every year in July. Specifically, children with paired annual serum samples demonstrating seroconversion or ≥4-fold increase in DENV-specific antibody titer, but who had not presented to the HCSFV with acute DENV illness, were considered to have experienced inapparent DENV infections [4],[8]. All confirmatory testing was conducted at the National Virology Laboratory (NVL) of the Nicaraguan Ministry of Health, the same laboratory responsible for confirmatory testing of routine, non-PDCS suspected dengue cases in Managua (see below). This study was approved by the Institutional Review Boards of the Nicaraguan Ministry of Health; the study hospital, Hospital Infantil Manuel de Jesús Rivera (HIMJR); the University of California, Berkeley; and the International Vaccine Institute. Parents or legal guardians of all subjects provided written informed consent, and subjects over 5 years of age gave verbal assent.

Between 2004 and 2008, a total of 4,742 children participated in the PDCS, with a yearly active cohort of 3,693-3,795 children with equal distribution by gender, age and neighborhood. Compliance and participation were consistently high [4]. Over 90% of participants sought medical care at the HCSFV at least once during the study period, and loss to follow-up – primarily due to moving outside of the study district – was low (4.3–7.1% annually). Possible dengue cases presented early to the HCSFV (94% presenting within the first 72 hours since symptom onset) and 94% provided a convalescent sample [4].

The National Epidemiologic Surveillance program (NES) collects information on suspected and confirmed dengue cases at the primary and secondary care level. Physicians in both the public and private sectors are required to report suspected cases meeting WHO criteria (see definition above) via a standardized MOH reportable disease form and to send blood samples (≥5 days since onset of fever) to the NVL at the MOH for testing. All febrile cases presenting at public facilities are reported, but only for those meeting the WHO definition is a suspected dengue report filled out and sample collected. Approximately 60% of suspected dengue cases in Managua have blood samples sent to the NVL (A. Balmaseda, unpublished data). A suspected case is considered positive (referred to here as a “confirmed” case) when the sample tests positive for DENV-specific IgM antibodies via IgM capture ELISA or, in rare cases, if the sample is obtained during the first 5 days after symptom onset and DENV RNA is detected.

Data on confirmed and suspected dengue cases by epidemiologic week among children 2–14 years old (and children 1–14 years old in 2004–5, before the NES began reporting 1 year-olds separately) were obtained from the NES for the years 2004–8, corresponding to the first four years of the PDCS. NES data include both confirmed and suspected cases reported by primary and secondary health facilities in Managua (primarily public, as private facilities do not comply as frequently with reporting guidelines), and are presented here by year, age group, and the health center corresponding to the district of residence of the child. The population of 1–14 year-olds (2004) and 2–14 year-olds (2005–8) in each health center's district was also obtained from the NES. These data were used to calculate incidence of confirmed dengue per 100,000 persons corresponding to each health center's district and overall in Managua. The expansion factor for confirmed cases was calculated by dividing the annual incidence of laboratory-confirmed symptomatic dengue among PDCS participants by the annual incidence of laboratory-confirmed dengue in Managua according to NES statistics. In addition, a sub-analysis was performed to calculate expansion factors using only data from 2–9 year-old children in both the PDCS and Managua (NES) in 2005–8 in order to compare the results with expansion factors calculated using the complete data sets, which could not be precisely age-matched (age-disaggregated data is not available from the NES: in 2004, NES dengue case reports are aggregated into 1–14 year-old age groups, and in 2005–8, into 1, 2–4, 5–9, and 10–14 year-old age groups). We also calculated an expansion factor for suspected dengue cases by dividing the annual incidence of suspected dengue cases in the PDCS meeting WHO criteria by the annual incidence of suspected dengue cases according to NES statistics. In addition, we calculated the ratio of inapparent DENV infections (defined above) to symptomatic dengue cases in the cohort. Annual incidence and expansion factors were calculated in relation to the annual dengue season, beginning in July of each year.

## Results

**Figure 1:**
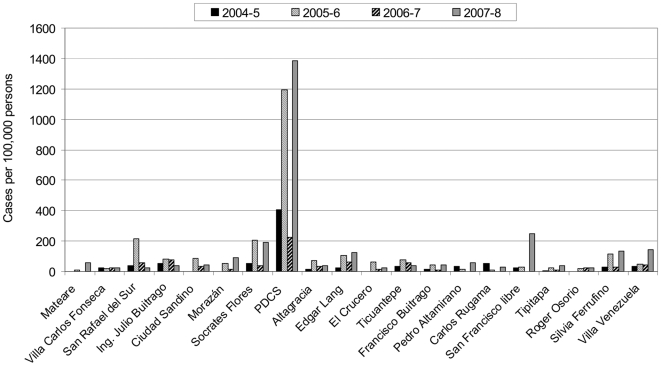
Reported laboratory-confirmed dengue cases among children in Managua health centers and in the PDCS, 2004–8. Laboratory-confirmed dengue cases among 1–14 year-old children in 2004–5 and 2–14 year-old children in 2005–8 reported in Managua health centers by the NES and in 2–12 year-old children meeting the WHO case definition in the PDCS 2004–8.

In the PDCS cohort, between 13 and 65 confirmed cases were recorded annually (Table 1), and between 0 and 51 confirmed cases were reported to the NES in individual health districts (data not shown). This translates to incidence of dengue ranging from 343 to 1,759 cases per 100,000 persons in the cohort study, as compared to 21 to 77 cases per 100,000 persons across all Managua's health centers (Table 1). The HCSFV, where the cohort study is based, reported greater numbers of confirmed and suspected dengue cases among its non-PDCS patients to NES than most other health centers (51 to 206 cases per 100,000, data not shown), though still only a fraction of the incidence observed in the cohort (Figure 1). The expansion factor ranged from 14 to 28 dengue cases in the cohort study for every confirmed case reported by the NES. Thus, despite year-to-year variation in the numbers of dengue cases, the cohort study consistently identified approximately 15- to 30-fold (average 21.3) more cases than were reported in Managua via national surveillance. Applying these annual expansion factors to national-level data, the estimated incidence of laboratory-confirmed dengue throughout Nicaragua ranged from approximately 300 to 1,000 cases per 100,000 persons. In a sub-analysis using only 2–9 year-olds in the PDCS and Managua NES data sets in 2005–8, we calculated expansion factors of 26, 19 and 21 – very similar to the numbers (24, 14 and 22) obtained using the full data sets in 2005–8. Expansion factors were also calculated based on PDCS and NES suspected dengue cases, yielding an average of 21 (range 16–28) times more suspected cases per 100,000 persons in the PDCS compared to those reported by NES throughout Managua.

**Table 1:** Incidence of confirmed and suspected dengue cases and expansion factors.

	2004–5	2005–6	2006–7	2007–8
**Laboratory-confirmed Symptomatic Dengue Cases**				
PDCS dengue cases (n)^1^	17	65	13	64
PDCS participants (n)^1,2^	3,721	3,695	3,795	3,693
PDCS dengue incidence per 100,000 persons	457	1,759	343	1,733
NES dengue cases reported in Managua health centers (n)^3^	99	252	102	278
Population of Managua in health center districts, excluding PDCS (n)^3^	464,733	398,079	405,378	358,824
Managua NES dengue incidence per 100,000 persons	21	63	25	77
** Expansion factor based on confirmed cases** 4	**21**	**28**	**14**	**22**
**Suspected Dengue Cases**				
PDCS suspected dengue cases (n)^5^	210	335	195	233
PDCS incidence of suspected dengue per 100,000 persons	5644	9066	5138	6309
Managua NES suspected dengue cases^6^	1384	1279	1116	1391
Managua NES incidence of suspected dengue per 100,000 persons	298	321	275	388
** Expansion factor based on suspected cases** 7	**19**	**28**	**19**	**16**
**Symptomatic and Inapparent DENV Infections in PDCS Cohort**				
Symptomatic DENV infections^8^	17	64	11	60
Inapparent DENV infections	276	318	176	182
** Ratio of inapparent to symptomatic DENV infections**	**16**	**5**	**16**	**3**
** Inapparent DENV infections for each symptomatic case reported by NES**	**347**	**138**	**218**	**68**

1 2–9 years old in 2004–5, 2–10 in 2005–6, 2–11 in 2006–7 and 2–12 in 2007–9 (the cohort aged by one year each year).

2 Includes all subjects active in the cohort for at least half of the study year, whether or not an annual sample was taken at the end of the year.

3 1–14 years old in 2004–5 and 2–14 years old for all other years.

4 Expansion factor  =  PDCS incidence of confirmed dengue/Managua NES incidence of confirmed dengue.

5 Includes suspected cases that met WHO criteria (see Materials and Methods); cases of undifferentiated fever, which account for 25% of laboratory-confirmed dengue cases in the PDCS, are not included in this figure.

6 Suspected dengue cases are defined by the NES according to WHO criteria (see Materials and Methods).

7 Expansion factor  =  PDCS incidence of suspected dengue/Managua NES incidence of suspected dengue.

8 Includes symptomatic cases identified only among those subjects who completed the study year and for which paired annual samples were available for determination of inapparent infection (n = 152, of 159 symptomatic dengue cases among cohort participants identified in 2004–8).

The ratio of inapparent to symptomatic DENV infection in PDCS participants also varied year-to-year [8] from 16 in 2004–5 and 2006–7, to 5 and 3 in 2005–6 and 2007–8, respectively (Table 1). Combining the ratio of inapparent to symptomatic DENV infection with the calculated expansion factor each year, we estimate 68–347 (average 193) inapparent DENV infections for every one symptomatic DENV infection reported to the NES. During the four years analyzed, 42% of inapparent DENV infections in the PDCS were primary, and thus at greater risk of more severe disease in a second DENV infection.

## Discussion

In this study, we have shown much higher incidence of symptomatic DENV infection in a pediatric cohort study than is reported to the national surveillance system from comparable urban public health centers. Annual expansion factor calculations indicate that up to 28 times more symptomatic DENV infections may occur than are reported to the NES, and under-reporting may be even greater among older age groups. This expansion factor, albeit a rough approximation, provides an estimate of the actual impact of dengue on this urban Latin American population, which may be of great use to governments and other institutions involved in dengue prevention and research. Furthermore, estimates of inapparent DENV infections and their immune status (primary vs. secondary) such as we provide here, especially when coupled with information about circulating DENV serotypes each year, may be useful for understanding the level of protective immunity in the population as well as help assess population risk for more severe dengue, since the single greatest risk factor for severe disease is a previous infection with a distinct DENV serotype [9]. This data also illustrates that total DENV transmission is distinct from transmission that can be observed clinically or reported through national surveillance systems.

This was not a classic capture-recapture study, but rather an ecological study comparing incidence rates in a cohort to national surveillance rates in the surrounding urban areas. The application and calculation of an expansion factor based on PDCS data has several limitations. The expansion factor cannot be applied to adult populations as only children, who are most affected by dengue in the study area, were included in the study. Limitations in NES reporting precluded calculations of incidence in precisely the same age groups as PDCS participants (e.g. 2–9 in 2004–5, 2–10 in 2005–6, etc); the larger NES population used (through 14 years old) may have led to slightly lower calculated expansion factors. However, in a sub-analysis restricted in both the PDCS and NES data to only 2–9 year-old children in 2005–8, expansion factors were found to be very similar to the numbers obtained with the full data set in 2005–6 and 2007–8 (26 vs. 28 and 21 vs. 22, respectively). The slightly higher expansion factor in 2006–7 for 2–9 year old children only (19 vs. 14) is due to the higher incidence that year in 10–14 year-olds in Managua; this was not apparent in the older PDCS participants, which included only 10 and 11 year-old children that year (Table 2). Additionally, the HCSFV district, which borders Lake Managua, may have higher dengue rates than other health centers, as there was more reported dengue among the non-study population of the HCSFV. However, another plausible explanation is that these higher numbers are due to the impact of the PDCS study protocol and increased awareness of dengue among both non-PDCS medical staff at the HCSFV and in the general population of the HCSFV catchment area. A greater expansion factor for pediatric dengue may be estimated in our study due to the inclusion of undifferentiated febrile illnesses that do not fit the traditional WHO definition, which account for approximately 25% of symptomatic cases identified in the cohort [8]. However, we also calculated an expansion factor based on suspected dengue cases (which only reflect the WHO criteria) in the PDCS compared to suspected cases in Managua health centers, and obtained very similar results as those calculated using only confirmed cases. While we were unable to restrict the estimates of national incidence to only urban areas due to limitations of the NES reporting system, since over 56% of Nicaragua is urban, using the entire Nicaraguan population could underestimate the expansion factor by up to 2-fold. However, all our calculated expansion factors (∼20-fold) are an order of magnitude greater than this potential correction factor; thus, we believe our overall estimates and conclusions to be valid and useful.

**Table 2:** Age-stratified incidence and expansion factors^1^.

	2005–6	2006–7	2007–8
**PDCS Incidence of Confirmed Dengue** 2			
Entire cohort (2–12 years old)	1,759	343	1,733
2–4 years old	1,469	412	1,173
5–9 years old	1,913	376	1,610
10–12 years old	1,749	143	2,734
**Managua NES Incidence of Confirmed Dengue** 2			
2–14 years old	63	25	77
2–4 years old	55	18	52
5–9 years old	74	21	83
10–14 years old^3^	57	33	87
**Expansion factors** 4			
2–14 years old	28	14	22
2–4 years old	27	23	22
5–9 years old	26	18	19
10–14 years old	30	4	31

1 NES data by age groups was not available for 2004–5; thus, analysis has been restricted to 2005–8.

2 Per 100,000 inhabitants.

3 Disaggregated data for 10, 11 and 12 year-old children is not available from the NES and thus the 10–14 year old age group has been used to calculate incidence and expansion factors.

4 Expansion factor  =  PDCS age-stratified incidence/Managua NES age-stratefied incidence.

Despite these limitations, our expansion factors falls in the same range as the only published expansion factors for ambulatory dengue. Meltzer et al. [10] calculated that 10 and 27 times more DENV cases occur in Puerto Rico than are reported in pediatric and adult populations, respectively. It is to be expected that expansion factors may vary somewhat in different countries with distinct surveillance programs, health care systems, and degrees of under-reporting. More sophisticated analysis is needed to calculate more precise expansion factors, for instance by controlling for socioeconomic and other district-level factors, and additional data is needed in order to calculate similar expansion factors for adult populations.

Nonetheless, an expansion factor such as we present here should allow for more accurate estimations of dengue burden and economic costs. Such estimates can benefit government surveillance programs, aid in allocation of resources to medical care and prevention, and facilitate calculation of the economic benefits of developing vaccines and drugs against dengue.
